# Comparison of DNA methylation profiles in human fetal and adult red blood cell progenitors

**DOI:** 10.1186/s13073-014-0122-2

**Published:** 2015-01-20

**Authors:** Samuel Lessard, Mélissa Beaudoin, Karim Benkirane, Guillaume Lettre

**Affiliations:** Montreal Heart Institute, 5000 Bélanger Street, Montréal, Québec H1T 1C8 Canada; Faculté de Médecine, Université de Montréal, 2900 Boul. Édouard-Montpetit, Montréal, Québec H3T 1J4 Canada; Hôpital Maisonneuve-Rosemont, 5415 Boul. de l’Assomption, Montréal, Québec H1T 2M4 Canada

## Abstract

**Background:**

DNA methylation is an epigenetic modification that plays an important role during mammalian development. Around birth in humans, the main site of red blood cell production moves from the fetal liver to the bone marrow. DNA methylation changes at the *β-globin* locus and a switch from fetal to adult hemoglobin production characterize this transition. Understanding this globin switch may improve the treatment of patients with sickle cell disease and β-thalassemia, two of the most common Mendelian diseases in the world. The goal of our study was to describe and compare the genome-wide patterns of DNA methylation in fetal and adult human erythroblasts.

**Methods:**

We used the Illumina HumanMethylation 450 k BeadChip to measure DNA methylation at 402,819 CpGs in *ex vivo*-differentiated erythroblasts from 12 fetal liver and 12 bone marrow CD34+ donors.

**Results:**

We identified 5,937 differentially methylated CpGs that overlap with erythroid enhancers and binding sites for erythropoiesis-related transcription factors. Combining this information with genome-wide association study results, we show that erythroid enhancers define particularly promising genomic regions to identify new genetic variants associated with fetal hemoglobin (HbF) levels in humans. Many differentially methylated CpGs are located near genes with unanticipated roles in red blood cell differentiation and proliferation. For some of these new candidate genes, we confirm the correlation between DNA methylation and gene expression levels in red blood cell progenitors. We also provide evidence that DNA methylation and genetic variation at the *β-globin* locus independently control globin gene expression in adult erythroblasts.

**Conclusions:**

Our DNA methylome maps confirm the widespread dynamic changes in DNA methylation that occur during human erythropoiesis. These changes tend to happen near erythroid enhancers, further highlighting their importance in erythroid regulation and HbF production. Finally, DNA methylation may act independently of the transcription factor *BCL11A* to repress fetal hemoglobin production. This provides cues on strategies to more efficiently re-activate HbF production in sickle cell disease and β-thalassemia patients.

**Electronic supplementary material:**

The online version of this article (doi:10.1186/s13073-014-0122-2) contains supplementary material, which is available to authorized users.

## Background

DNA methylation is a dynamic epigenetic mark mostly found on cytosine residues of certain CpG dinucleotides in mammalian genomes. In humans, it is involved in gene imprinting, X-chromosome inactivation, and transposable element suppression [1]. DNA methylation is generally associated with transcriptional silencing and plays an essential role in maintaining stem cell pluripotency and controlling other cell- or organ-specific developmental programs [1]. Despite the well-established role of DNA methylation during development, we are only now starting to collect single-base resolution maps of these developmentally regulated DNA methylation changes thanks to improvements in DNA array- and sequencing-based technologies [2]. Such precise DNA methylome maps are important in order to understand how transcriptional networks are controlled during development or how dys-regulation of DNA methylation - observed in human diseases like cancer or upon treatment with DNA demethylating agents - may impact cell functions and/or identity [3].

Characterization of mouse models with conditional deletions of genes that encode DNA methyltransferases - enzymes that catalyze the transfer of methyl groups onto DNA - has firmly established that DNA methylation is essential during hematopoietic stem cell differentiation and repopulation [4,5]. Recent reports used enrichment of methylated DNA followed by next-generation DNA sequencing to characterize genome-wide DNA methylation changes in murine hematopoietic stem and progenitor cells, and during erythropoiesis [6,7]. The studies showed a progressive DNA demethylation during erythroid differentiation, and confirmed that groups of CpGs in promoters - so-called CpG islands - tend to be hypomethylated and correlated with active gene expression [6,7].

Although large-scale projects are starting to generate comprehensive DNA methylation datasets [8], they do not capture all stages of human erythroid differentiation and proliferation. In an initial study, investigators monitored genome-wide changes in DNA methylation during human erythropoiesis of bone marrow-derived CD34+ progenitor cells and reported global DNA hypomethylation [9]. We are particularly interested in the epigenetic differences between fetal- and adult-stage erythroblasts that originate, respectively, from the fetal liver and the bone marrow. The large epigenomic projects (e.g. ENCODE, Roadmap Epigenomic, Blueprint) do not profile DNA methylation in these erythroid cells. During this erythroid transition, which occurs around birth in humans, erythroblasts reduce the production of fetal hemoglobin (HbF) and increase the production of adult hemoglobin (HbA) through the transcriptionally regulated fetal-to-adult hemoglobin switch [10]. This gene expression switch is accompanied by progressive DNA hypermethylation of the *HBG2* promoter, which encodes the γ-globin subunit of HbF [11,12]. Understanding the molecular mechanism behind the fetal-to-adult hemoglobin switch is particularly important since re-activating HbF production is the most promising therapy for patients with sickle cell disease and β-thalassemia [10]. *Ex vivo* differentiation protocols now exist to cultivate sufficient number of fetal and adult human erythroblasts to extend the characterization of DNA methylation to the rest of the genome [13].

Here we provide comprehensive DNA methylome maps of human erythroblasts differentiated *ex vivo* from CD34+ progenitor cells purified from the fetal liver or the bone marrow. By analyzing DNA methylation values at 402,819 tested CpGs in fetal and adult erythroblasts, we identified 5,937 differentially methylated CpGs. These differentially methylated regions include the known *β-globin* locus, other genes with known roles in erythropoiesis, as well as several other genes with no previously recognized functions in red blood cell differentiation. We showed that the differentially methylated CpGs cluster within stage-specific erythroid enhancers and are located near binding motifs for transcription factors that regulate hematopoiesis. Finally, we determined that DNA methylation and genetic variation at the *β-globin* locus independently control HbF production in adult-stage cells.

## Methods

### Cell culture and differentiation

Primary human fetal and adult CD34+ hematopoietic stem/progenitor cells harvested from 24 anonymous donors (12 of each) were purchased from DV Biologics [14] and Lonza [15], respectively. Primary fetal and adult erythroblasts were generated using two-phase serum-free culture as described previously [13]. Briefly, primary human fetal and adult CD34+ cells are cultured in the expansion medium until day 6. They are then transferred to the differentiation medium until cells are collected at day 18. The medium is changed every 3 days. We assessed cell morphology by Wright-Giemsa coloration, and measure cell size and count using the MOXI Z Mini automated cell counter (ORFLO technologies, ID), and hemoglobin production by capillary electrophoresis.

### Genomic DNA extraction and methylation assay

We extracted genomic DNA with the Gentra Puregene Cell Kit (Qiagen). DNA was further precipitated with alcohol in order to obtain highly purified DNA. DNA bisulfite conversion was performed using the EZ DNA Methylation Gold Kit (Zymo Research, CA, USA). We used the Infinium HumanMethylation450 BeadChip (Illumina Inc., CA, USA) to measure genome-wide patterns of DNA methylation; the experiment was carried out at the Genome Quebec-McGill Innovation Centre using Illumina’s recommended protocol. We assessed data quality with the minfi R package [16] and normalized intensities using the ARRm software, which corrects for probe type, background, dye, and position effects [17]. We removed probes that target a genomic sequence annotated to carry genetic variants based on dbSNP version 137 (N = 82,694). DNA methylation data have been submitted and are available from the National Center for Biotechnology Information (NCBI) Gene Expression Omnibus (GEO) repository (accession number (GEO: GSE56491)). To identify single CpG that are differentially methylated between fetal and adult erythroblasts, we converted DNA methylation β-values into M-values [18] and used linear regression in R, correcting for sex effects. To combine results across CpGs located within a functional genomic unit, we used a generalization of Fisher’s method that takes into account correlation between nearby CpGs and that is already implemented in the RnBeads software [19]. We used RefSeq (release 61) coordinates to map CpGs to specific genes. Promoter CpGs are located within 1.5-kb upstream of a RefSeq gene and enhancer CpGs map to erythroid enhancers experimentally identified in *ex vivo* differentiated CD34+ cells [20]. In total, we could map 233,894 CpGs to 25,891 gene bodies, 130,854 CpGs to 25,103 promoters, and 11,709 CpGs to 4,604 erythroid enhancers. DNA methylation levels at the *HBG2* promoter were measured using the MassArray EpiTYPER platform (Sequenom Inc., CA, USA), which quantitatively analyze fragmented PCR products using MALDI-TOF mass spectrometry. Bisulfite-converted DNA was amplified by PCR as previously described [21].

### RNA extraction and gene expression analysis

RNA was extracted using the RNeasy Plus Mini Kit from Qiagen. We determined the quality and quantity of RNA using the RNA 6000 Nano kit on the Bioanalyzer instrument (Agilent). We converted RNA into cDNA with the High Capacity cDNA Reverse Transcription Kit (ABI) and performed quantitative PCR using the Platinum SYBR Green qPCR Mix (Invitrogen). Relative gene expression levels were measured using the ΔΔC_t_ method and we evaluated statistical significance with *t*-test as implemented in the R software (v. 3.0.0). Primer sequences are listed in Additional file 1: Table S1. Total RNA for RNA sequencing was extracted using the miRNEASY kit (Qiagen). Paired-end RNA sequencing was performed on the Illumina Hiseq 2000 platform. Reads were mapped to the genome using Tophat2 (v.2.0.9) and transcript abundances were estimated using Cufflinks (v.2.2.1).

### Enrichment analyses

We used binomial tests to measure the enrichment of differentially methylated CpGs in gene bodies, promoters, and erythroid enhancers as compared to the rest of the genome surveyed by the Illumina HumanMethylation450 BeadChip. To compare the enrichment of differentially methylated CpGs in erythroid enhancers versus other enhancers present in different cell types, we obtained enhancer coordinates from nine cell lines analyzed by the ENCODE Project [22]. We used DAVID to measure the enrichment of gene ontology (GO) terms and biological pathways among genes with a mean β-value difference >0.1 and a combined *P* <9 × 10^−7^ in their body or promoter, taking into account the coverage of the Illumina HumanMethylation450 BeadChip [23,24]. To identify transcription factor binding motifs that are enriched at differentially methylated loci, we used the HOMER software and a list of 495 pre-defined transcription factor motifs [25], limiting the search to 200 base pairs on both sides of differentially methylated CpGs. For this analysis, we compared the 5,000 most hypomethylated regions in fetal erythroblasts to the 5,000 most hypomethylated regions in adult erythroblasts, as recommended. HOMER results were stable using different thresholds to select hypomethylated regions.

### DNA genotyping and association studies

Genotyping was performed at the Pharmacogenomics Center of the Montreal Heart Institute using the HumanOmniExpress BeadChip array (Illumina). We performed quality-control steps with PLINK [26], removing markers with genotyping success <90% or Hardy-Weinberg *P* <1.0 × 10^−6^. All samples had a genotyping success rate >99.8%. We imputed genetic variants with the MaCH/minimac software [27] and reference haplotypes from the 1000 Genomes Project (phase I). For association analysis, we only considered genotyped markers or markers with an imputation quality r2_hat >0.6. We tested association between SNP genotypes and methylation levels under an additive model using linear regression as implemented in R. For *BCL11A*, we tested CpGs inside the gene body or the promoter (chr2:60,678,302-60,781,633). For *HBS1L-MYB*, tested CpGs were either inside the gene bodies and promoters of the genes, or in the intergenic region (chr6:135,281,517-135,540,311). For the *β-globin* locus, all CpGs between the promoter of *HBE* and the gene body of *HBB* were included (chr11:5,246,696- 5,527,882).

To test the enrichment of HbF association signals with DNA methylation-implicated erythroid enhancers, we accessed genome-wide association study (GWAS) data of 1,140 African Americans from the Cooperative Study of Sickle Cell Disease (CSSCD). These individuals were genotyped on the Illumina Human610-Quad array, as previously described [28]. SNPs were phased using MaCH (v1.0.16) and imputed with haplotypes from European and African samples generated by the 1000 Genomes Project (phase I) using minimac (v4.4.3). Association *P* values with HbF were calculated using mach2qtl (v1.0.8). In total, 6,994,357 SNPs were included in the analysis. We found 63,876 SNPs that overlap with 12,683 erythroid enhancers.

## Results and discussion

### *Ex vivo* culture of erythroid progenitor cells

We used a previously established two-phase cell culture protocol to expand and differentiate primary human fetal and adult CD34+ hematopoietic stem/progenitor cells into erythroblasts [13]. For each tissue (fetal liver or bone marrow), we differentiated CD34+ cells from 12 anonymous donors to compare not only DNA methylation changes between fetal and adult erythroblasts, but also between individuals within a developmental stage. We validated the expansion and differentiation cell culture protocol by characterizing cell size, growth, and morphology (Additional file 1: Figure S1). To determine if erythroblasts differentiated *ex vivo* maintain characteristics that are specific to their tissue of origin, we measured expression of genes involved in the fetal-to-adult hemoglobin switch and quantified hemoglobin production by capillary electrophoresis. As expected, erythroblasts derived from bone marrow express significantly more *BCL11A* and *KLF1* than fetal erythroblasts (Additional file 1: Figure S2A-B). *BCL11A* and *KLF1* are transcriptional repressors of *HBG2* gene expression; *KLF1* also increases *HBB* gene expression, which encodes the β-globin subunit of HbA [13,29-31]. Consistently, erythroblasts from the fetal stage produce exclusively HbF (100 ± 0%) whereas differentiated cells from the adult stage produce mostly HbA (84.2 ± 4.8%) (Additional file 1: Figure S2C-E). Using a targeted assay that measures DNA methylation at seven CpGs within the *HBG2* promoter, we also confirmed hyper- and hypomethylation of this promoter in adult and fetal erythroblasts, respectively (Additional file 1: Figure S3) [32]. Taken together, these results confirm that the erythroblasts have maintained their developmental stage specificity.

In the mouse and humans, it is known that global demethylation occurs during erythropoiesis [6,7,9]. For this reason, it is important to confirm that fetal and adult erythroblasts grow and differentiate under similar kinetics. To directly address this concern, we performed a time-course analysis of the expression of genes that code for markers of differentiation and that are localized at the cell surface of erythroblasts: *CD34*, *CD71* (transferrin receptor (*TRFC*)), and *CD235a* (glycophorin A (*GYPA*)). Recent transcriptomic analyses in *ex vivo*-differentiated human erythroblasts have confirmed that the expression levels of these genes are strongly correlated with the localization of the encoded proteins at the cellular membrane [33,34]. As expected, *CD34* is highly expressed at the beginning of differentiation and decreases thereafter, whereas the expression of *CD71* and *CD235a* increases during differentiation (Additional file 1: Figure S4). At any given time point, the expression of these three genes was not different between fetal liver- and bone marrow-derived CD34+ progenitors cells, except for *CD235a* at the beginning of differentiation (more expressed in fetal liver cells) (Additional file 1: Figure S4). These results confirm that fetal and adult erythroblasts grow and differentiate at a similar rate, and therefore that our experiment is adequate to detect differences in DNA methylation that are mostly due to the different developmental stage (fetal liver vs. bone marrow) of these cells.

### The DNA methylation landscape in human erythroblasts

We used the Illumina HumanMethylation 450 k BeadChip to measure quantitatively DNA methylation across the genome in *ex vivo*-differentiated erythroblasts from 12 fetal liver and 12 bone marrow donors. After quality control and intensity normalization steps, we obtained DNA methylation β-values (from 0.0 (unmethylated) to 1.0 (fully methylated)) for 402,819 CpGs (Additional file 1: Figure S5). We only used these CpGs in subsequent analyses. We used DNA methylation data generated by the Roadmap Epigenomics Project [35] using reduced representation bisulfite DNA sequencing (RRBS) from mobilized adult CD34+ cells to validate the DNA methylation levels measured in erythroblasts with the Illumina HumanMethylation 450 k BeadChip. Although not differentiated *ex vivo*, we reasoned that these CD34+ cells would be a good proxy for adult erythroblasts used in our experiment. Across 80,136 CpGs available in both datasets, we observed a strong correlation of DNA methylation values (Pearson’s *r* = 0.91, *P* <2.2 × 10^−16^, Additional file 1: Figure S6A). Importantly, we repeated the same analysis with other RRBS datasets from the Roadmap Epigenomics Project. We observed that DNA methylation values in erythroblasts are more correlated with DNA methylation data from blood-related rather than blood-unrelated cells or tissues (Additional file 1: Figure S6B). These comparisons confirm the quality and specificity of the DNA methylation data generated with the Illumina 450 k array in human erythroblasts.

**Figure 1:**
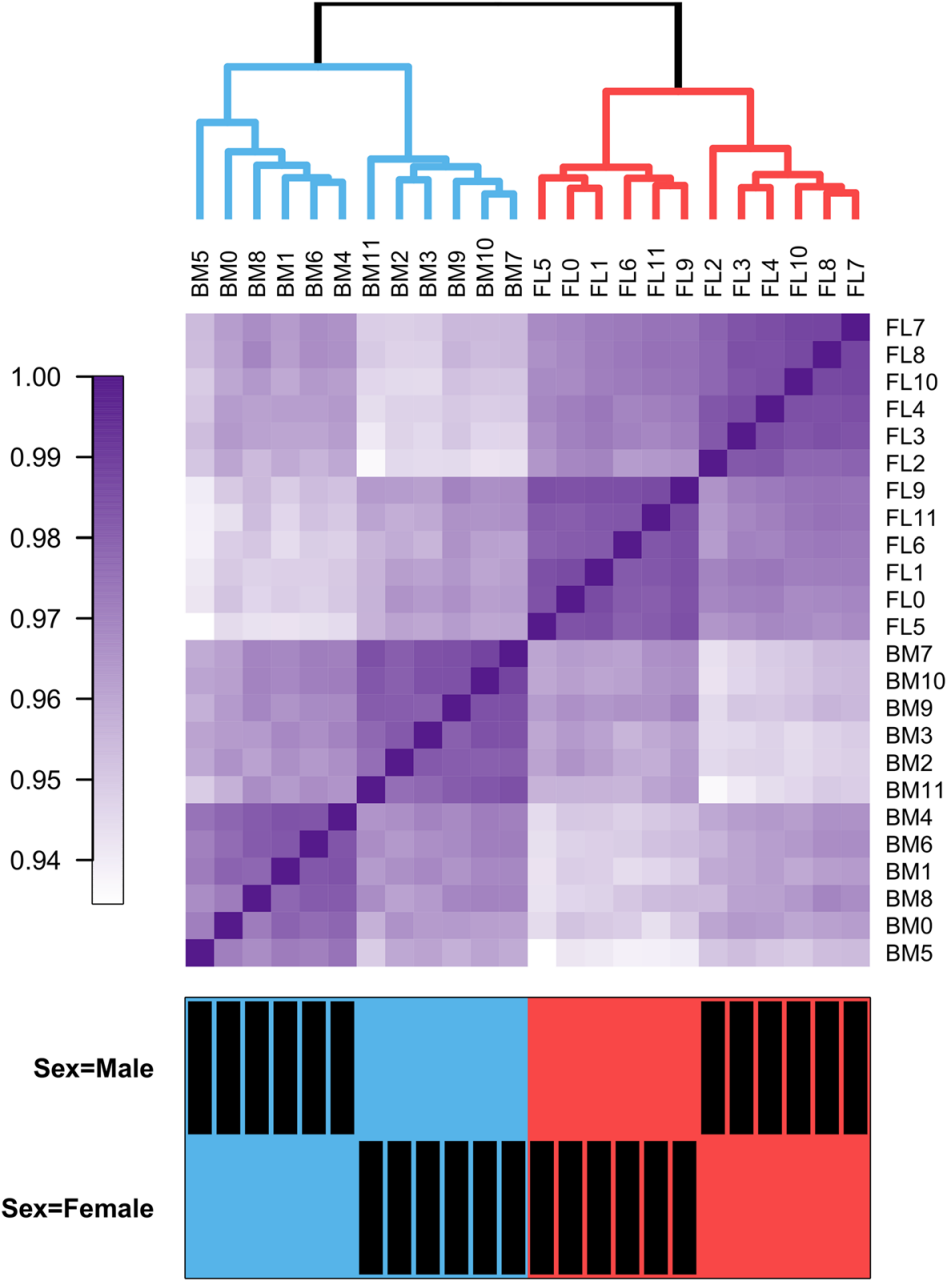
**Hierarchical clustering analysis of DNA methylation in erythroblasts.** Unsupervised clustering analysis of DNA methylation at 402,819 CpGs separates precisely fetal liver-derived from bone marrow-derived erythroblasts. Within each developmental stage, cells are sub-divided by the sex of the donors. The top panel represents the dendogram of the different clusters identified. The middle panel summarizes pairwise correlations between all samples. In the bottom panel, black rectangles identify male and female donors. BM: adult erythroblasts; FL: fetal erythroblasts.

We analyzed DNA methylation across all CpGs tested using unsupervised clustering methods. The two main clusters accurately distinguish fetal liver- from bone marrow-derived erythroblasts (Figure 1). This result confirms our hypothesis that changes in DNA methylation captures developmental differences between fetal and adult erythroblasts. We also noted that the two main stage-specific clusters are sub-divided according to the sex of the sample’s donors (Figure 1). This sex-specific clustering is mostly dependent on DNA methylation values at CpGs located on the X-chromosome.

### Differential DNA methylation between fetal and adult erythroblasts

**Figure 2:**
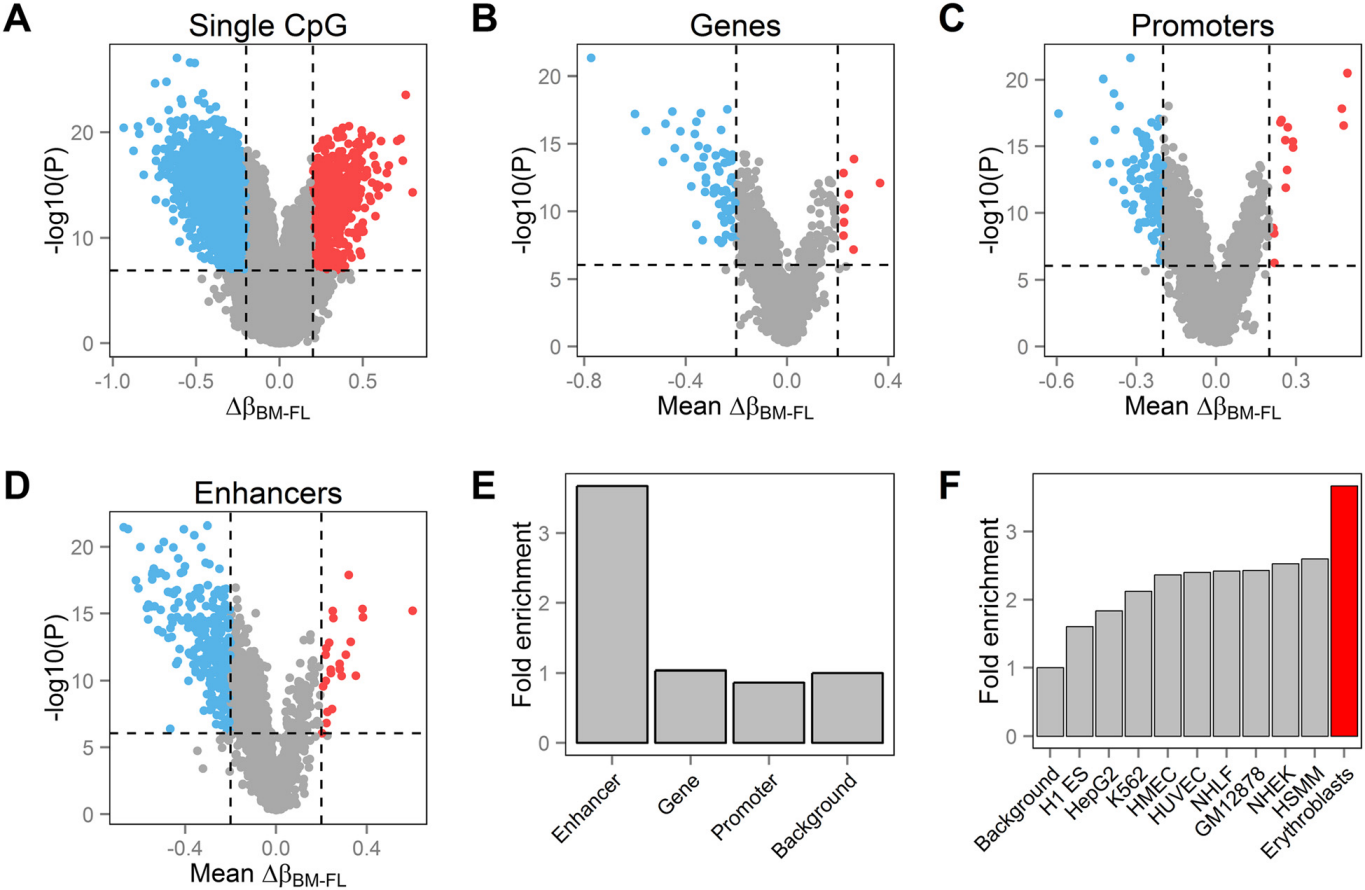
**Differential DNA methylation between fetal liver- and bone marrow-derived erythroblasts.** Volcano plots of differentially methylated **(A)** CpGs, **(B)** genes, **(C)** promoters, and **(D)** erythroid enhancers. Differentially methylated single CpGs have a difference in DNA methylation β-values between fetal and adult erythroid cells ≥20% and a *P* ≤1.25 × 10^−7^. Red and blue points correspond to CpGs significantly hypomethylated in fetal- and adult-stage erythroblasts, respectively. For genes, promoters, and erythroid enhancers, we combined *P* values from CpG sites that fall within each unit using a generalization of Fisher’s method to take into account correlation of values at nearby sites. For genes, promoters, and enhancers, we averaged the DNA methylation β-values of the CpGs that fall within the unit (*y*-axis). We used a Bonferonni-adjusted statistical threshold to define differentially methylated functional regions. **(E)** An enrichment of differentially methylated CpGs was observed in erythroid enhancers when compared to gene or promoter regions also tested on the Illumina HumanMethylation 450 k BeadChip. We used probes that fall outside of these regions to calculate the fold enrichment. **(F)** Erythroid enhancers (red) are enriched in differentially methylated CpGs when compared to enhancers defined in other cell lines using data from the ENCODE Project.

We sought to identify differentially methylated single CpG sites by comparing methylation β-values for fetal and adult erythroblasts, taking into account potential sex effect. We used two criteria to define differentially methylated CpGs: a difference in β-values of ≥0.2 and a *P* ≤1.25 × 10^−7^ (significant threshold after Bonferonni correction). Using this definition, we found 5,937 differentially methylated CpGs (Figure 2A and Additional file 2: Table S2).

**Figure 3:**
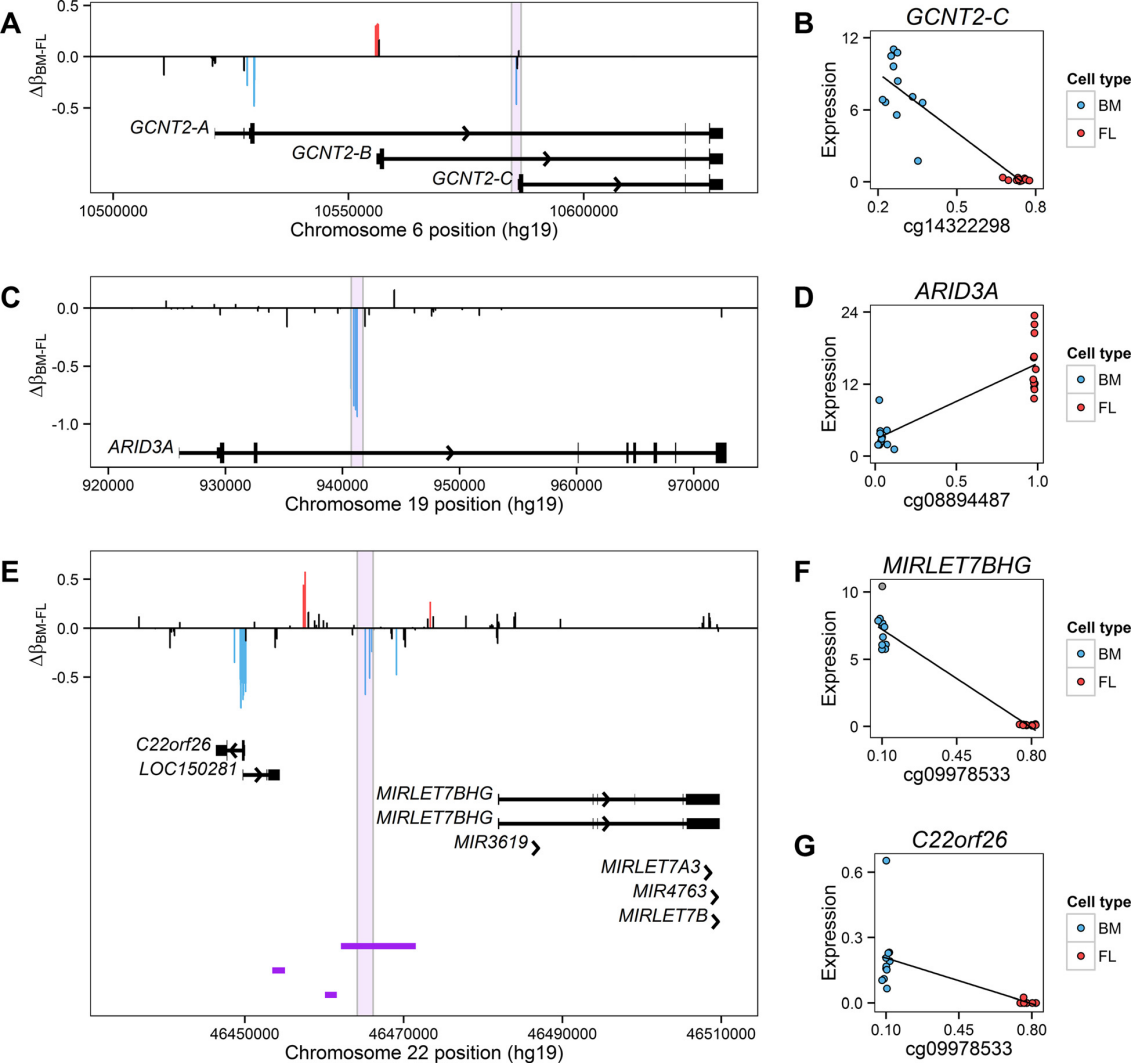
**Examples of loci differentially methylated between fetal- and adult stage erythroblasts.** Each vertical line represents a targeted CpG. Red and blue lines correspond to CpGs significantly hypomethylated in fetal- and adult-stage erythroblasts, respectively (*P* <1.25 × 10^−7^ and Δβ ≥0.2). **(A)** A CpG in the promoter of *GCNT2* isoform C, which is responsible for the conversion of the fetal i-antigen to the adult I-antigen, is hypomethylated in adult-stage erythroblasts. **(B)** *GCNT2-C* expression levels is inversely correlated with DNA methylation at a CpG located in its core promoter (*r* = −0.97, *P* = 4.6 × 10^−14^). **(C)** CpGs in an intron of *ARID3A*, a gene implicated in hematopoiesis, displays hypomethylation in adult-stage erythroblasts. **(D)** *DNA* methylation inside *ARID3A* (highlighted) positively correlates with its expression levels (*r* = 0.87, *P* = 9.6 × 10^−8^). **(E)** Differential DNA methylation near *C22orf26* and the miRNA *let7-b* host gene (*MIRLET7BHG*). A cluster of hypomethylated CpGs in adult erythroblasts overlaps an active erythroid enhancer (purple horizontal bars). DNA methylation inside the enhancer (highlighted) negatively correlates with **(F)** *MIRLET7BHG* (*r* = −0.99, *P* = 5.6 × 10^−20^) and **(G)** *C22orf26* expression levels (*r* = −0.74, *P* = 3.2 × 10^−5^).

We discuss below two interesting examples of differentially methylated loci detected in human erythroblasts. *GCNT2* encodes the enzymes responsible for the conversion of the blood i- to the I-antigen during the fetal-to-adult transition in erythroblasts. *GCNT2* codes for three isoforms: isoform C is responsible for the I-antigen phenotype in adult erythrocytes [36]. In our cell culture system, we found that cg14322298 in the promoter of isoform C is hypomethylated in adult erythroblasts (Δβ_adult-fetal_ = −0.47, *P* = 8.0 × 10^−16^; Figure 3A). We found that *GCNT2-C* expression levels correlate with DNA methylation at cg14322298 in erythroblasts (Figure 3B). Isoform B had very low expression in both fetal and adult erythroblasts, whereas isoform A and C explained most of *GCNT2* expression in fetal (72.2%) and adult (75.1%) erythroblasts, respectively (Additional file 1: Figure S7). Another example of differentially methylated loci include CpGs in the *ARID3A*/*Bright* gene that show a marked differential DNA methylation pattern, being almost completely methylated in fetal-stage cells (e.g. cg08894487, Δβ_adult-fetal_ = −0.93, *P* = 3.8 × 10^−21^) (Figure 3C). This gene is required for hematopoietic stem cell development: definitive erythrocyte formation in the fetal liver is impaired in *Bright* knockout mice, but not at the primitive (embryonic) stage [37]. *ARID3A* expression was higher in fetal erythroblasts (Figure 3D). Positive correlations between DNA methylation inside gene bodies and gene expression levels have been reported previously [38]. Overall, we observed a global enrichment of positive correlations between DNA methylation at CpGs inside gene bodies and expression levels in erythroblasts. In contrast, there is a marked enrichment of negative correlations between CpGs within promoters or enhancers, and gene expression (Additional file 1: Figure S8).

### Erythroid enhancers are enriched for differentially methylated CpGs

To link changes in DNA methylation to biologically relevant functional units, we combined results from single CpGs that fall within each of 25,891 transcripts defined using RefSeq, 25,103 promoters (1.5-kb upstream of RefSeq transcripts) and 4,604 erythroid enhancers [20]. These enhancers, defined using DNase I hypersensitive sites and histone marks, include a set of enhancers that is common to both fetal and adult erythroblasts, as well as developmental lineage-specific enhancers [20]. After Bonferonni correction (significance threshold set at *P* <9 × 10^−7^), we found 77 genes, 116 promoters, and 303 erythroid enhancers that are differentially methylated (Figure 2B-D and Additional file 3: Table S3).

These analyses highlighted multiple regions of interest (Additional file 3: Table S3). For instance, *C22orf26* was one of the most differentially methylated genes (mean Δβ_adult-fetal_: −0.60; *P* = 6.2 × 10^−18^). Although nothing is known about this gene, it is located just upstream of the microRNA *let7-b* host gene (*MIRLET7BHG*; Figure 3E). The microRNA *let-7b* belongs to the *let-7* family, is highly expressed in adult as compared to fetal erythroblasts, and correlates with *BCL11A* expression and reduced HbF levels [39]. It is possible that DNA methylation at CpGs within or near *C22orf26* regulates the expression of *MIRLET7BHG*. Notably, a nearby enhancer active in adult-stage erythroblasts contain differentially methylated CpGs, which correlates with *MIRLET7BHG* expression (Figure 3F). Although the CpGs also correlates with *C22orf26* expression levels (Figure 3G), this gene is an order of magnitude less expressed, and other CpGs in its core promoter might also control its expression. Finally, we carried out a pathway-based analysis to identify group of functionally related genes that are differentially methylated. We found multiple pathways enriched for signaling activity, wound healing, oxygen, cytokine production, circulation, and cation transport (Additional file 4: Table S4 and Additional file 1: Table S5). Many of these pathways include genes not associated with erythropoiesis previously, and we need to validate if differential DNA methylation translates into biologically important functions.

More generally, we asked whether there was an enrichment of differentially methylated CpGs in genes, promoters, and erythroid enhancers when compared to the rest of the genome tested by the Illumina HumanMethylation 450 k BeadChip. We observed a strong statistically significant enrichment of differentially methylated CpGs in erythroid enhancers (3.67-fold, *P* = 9.5 × 10^−164^) (Figure 2E). The enrichment was marginally significant in genes (1.03-fold, *P* = 0.04) and we noted a significant depletion of differentially methylated CpGs in promoters (0.86-fold, *P* = 4.3 × 10^−10^) (Figure 2E). The results for promoters and genes are challenging to interpret given the ascertainment bias in the design of the methylation array. However, this technical confounding does not affect the result for the erythroid enhancers since the release of the Illumina HumanMethylation 450 k BeadChip pre-dated the publication of the erythroid enhancers [20]. Therefore, differential DNA methylation at erythroid enhancers likely captures transcriptional and developmental differences between fetal and adult erythroblasts.

We then compared enrichment of differentially methylated CpGs in enhancers defined in nine different cell types using data from the ENCODE Project [22]. Although there was enrichment at enhancers from other cell types - perhaps suggesting constitutive regulatory functions - we observed the strongest enrichment for erythroid enhancers defined in differentiated CD34+ cells (significant against enhancers from all other cell types, *P* <4.5 × 10^−17^, Figure 2F) [20]. The enrichment of differentially methylated CpGs within enhancers defined by ENCODE in erythroleukemic K562 cells was similar to the enrichment observed in other, non-hematopoietic cells (Figure 2F). This result may highlight a limitation in using K562 cells to study the fine regulatory mechanisms that control erythropoiesis.

**Figure 4:**
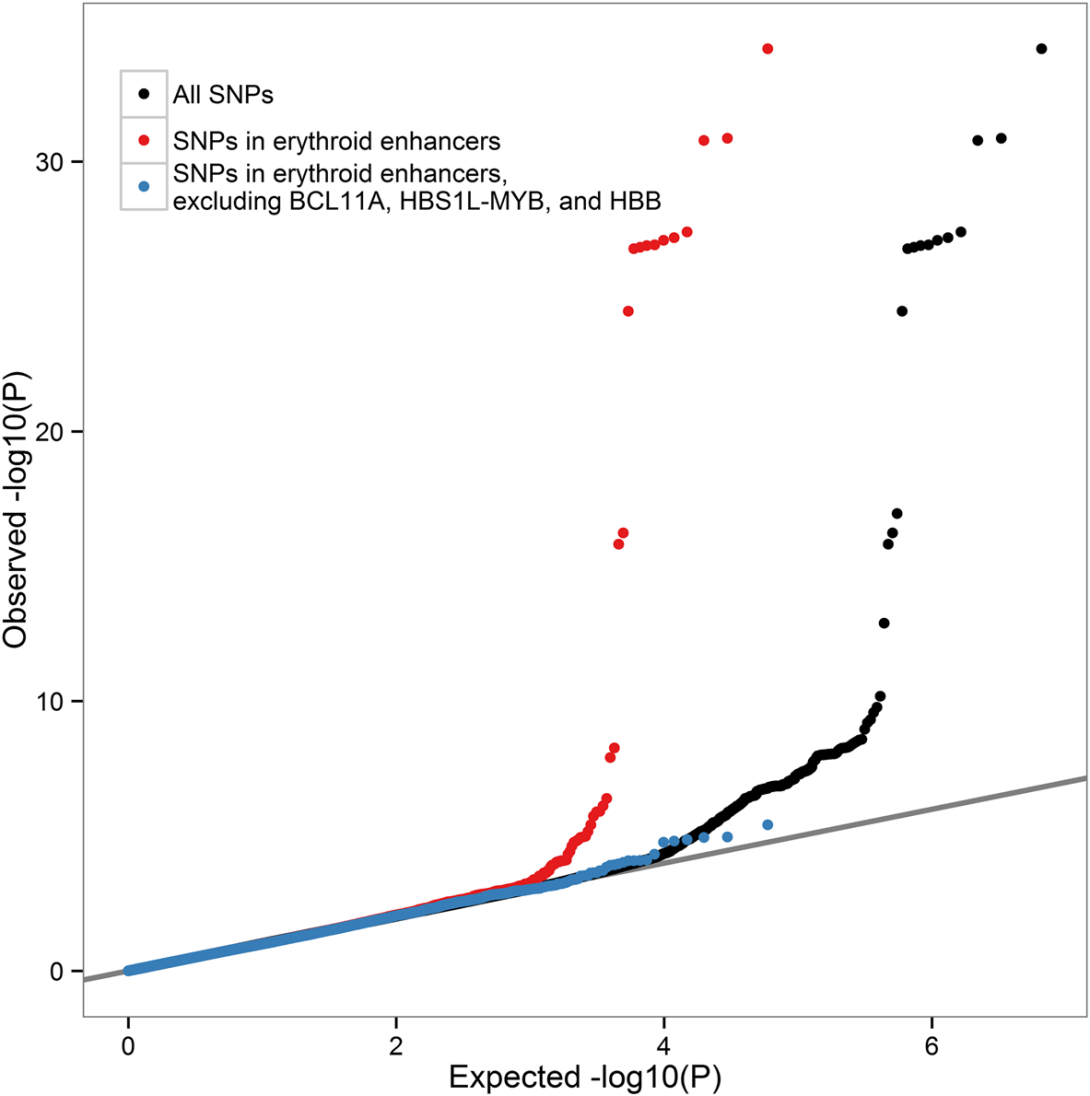
**Erythroid enhancers are enriched for SNPs associated with fetal hemoglobin (HbF) levels in patients with sickle cell disease.** Quantile-quantile (QQ) plot of association *P* values with HbF levels in 1,140 sickle cell disease patients. The QQ plot for all 6,994,357 imputed SNPs is shown in black (λ_GC_ = 1.01). In red are the 63,876 markers that map to erythroid enhancers (λ_GC_ = 1.03) and in blue are the markers that remain after excluding enhancers near *BCL11A*, *HBS1L-MYB* and the *β-globin* locus (λ_GC_ = 1.02).

Having demonstrated an enrichment of differentially methylated CpGs in erythroid enhancers, we were interested in testing if these genomic regions are more likely to contain genetic variants associated with HbF levels. We analyzed associations between HbF levels measured at baseline and genotypes at 6,994,357 markers in 1,140 adult patients with sickle cell disease [28]. As expected, we observed a strong deviation from the null distribution owing to variants in the *BCL11A*, *HBS1L-MYB*, and *β-globin* loci (Figure 4). When we considered only the 63,876 SNPs that overlap with erythroid enhancers, we again observed deviation from the null expectation. This departure from the null was even true - although more modest - after excluding enhancers near the known HbF regulators (Figure 4). This result directly implicates erythroid enhancer in inter-individual HbF levels variation, and suggests that future genetic experiments to find new HbF regulators should focus on these genomic regions.

### Several transcription factor binding motifs are preferentially located near differentially methylated CpGs

Data from the ENCODE Project support a passive role for DNA methylation in controlling gene expression: when a transcription factor is not or less expressed, the CpGs surrounding its consensus binding sites tend to be less accessible to DNase I digestion and to be more methylated [40]. Based on this observation, we reasoned that an analysis of transcription factor binding sites within loci that are differentially methylated between fetal and adult erythroblasts might yield new transcription factors important for erythroid development. We performed this analysis on the 5,000 most hypomethylated CpGs in fetal erythroblasts and compared them to the 5,000 most hypomethylated CpGs in adult erythroblasts. We obtained consistent results when using different thresholds. We included 200 base pairs on each side of the CpGs and considered 495 transcription factor binding motifs defined by the HOMER software [25]. We identified enrichment for many transcription factors involved in different cellular processes, including many with known roles during erythropoiesis (Additional file 1: Tables S6-S7). In Table 1, we list the top transcription factors with binding motifs enriched near hypomethylated CpGs.

**Table 1:** **Enrichment of transcription factor binding sites (TFBS) near differentially methylated CpGs**

**Motif name**	**Consensus**	***P*** **value**	***q*** **-value**	**Fold enrichment**
*TFBS enriched near CpGs hypomethylated in fetal erythroblasts*				
SOX2	BCCATTGTTC	1.0 × 10^−13^	0	1.5
TCF3	ASWTCAAAGG	1.0 × 10^−8^	0	1.8
REST-NRSF	GGMGCTGTCCATGGTGCTGA	1.0 × 10^−8^	0	5.5
SOX6	CCATTGTTNY	1.0 × 10^−8^	0	1.3
GATA1	AGATGKDGAGATAAG	1.0 × 10^−5^	0.0001	2.4
MAZ	GGGGGGGG	1.0 × 10^−5^	0.0002	1.1
TCF4	ASATCAAAGGVA	1.0 × 10^−5^	0.0002	1.4
FOXA1	WAAGTAAACA	1.0 × 10^−5^	0.0002	1.3
HNF6	NTATYGATCH	1.0 × 10^−4^	0.0004	1.4
TCFL2	ACWTCAAAGG	1.0 × 10^−4^	0.0005	1.9
*TFBS enriched near CpGs hypomethylated in adult erythroblasts*				
NF1	CYTGGCABNSTGCCAR	1.0 × 10^−211^	0	3.9
IRF2	GAAASYGAAASY	1.0 × 10^−78^	0	7.3
TLX/NR2E1	CTGGCAGSCTGCCA	1.0 × 10^−76^	0	2.4
NF1-halfsite	YTGCCAAG	1.0 × 10^−56^	0	1.4
ISRE	AGTTTCASTTTC	1.0 × 10^−54^	0	7.5
BACH1	AWWNTGCTGAGTCAT	1.0 × 10^−42^	0	6.4
RUNX1	AAACCACARM	1.0 × 10^−37^	0	1.6
RUNX2	NWAACCACADNN	1.0 × 10^−36^	0	1.7
NRF2	HTGCTGAGTCAT	1.0 × 10^−36^	0	5.8
c-JUN	GATGASTCATCN	1.0 × 10^−33^	0	2.3

For these analyses, we used the HOMER software and analyzed TFBS located near CpGs (±200 base pairs) that are hypomethylated in fetal or adult erythroblasts. The top 10 enriched motifs are shown here for each cell type; see Additional file 1: Tables S6-S7 for the complete list of significant TFBS. We calculated *q*-values using the Benjamini-Hochberg method. We calculated the fold enrichment by comparing the number of hypomethylated CpGs near a given TFBS in fetal and adult erythroblasts. The consensus motif follows the IUPAC nomenclature when more than one base is possible.

In fetal erythroblasts, we observed enrichment for the binding sites of SOX6 and GATA1, two key transcription regulators of hematopoiesis [10,41,42]. GATA binding motifs are also enriched at fetal-specific erythroid enhancers [20]. GATA1 and SOX6 are two transcription factors important in fetal and adult erythroblasts, although our results suggest that they may preferentially bind hypomethylated sites in fetal erythroblasts [43]. The motif recognized by NFY was enriched near CpGs hypomethylated in fetal erythroblasts: NFY binds the *HBG2* gene promoters to stimulate chromatin opening [44]. In adult erythroblasts, we detected an enrichment for the motif bound by NF-E2, a transcription factor important for erythroid maturation and *HBB* gene expression [45], as well as RUNX1, an important regulator of mammalian hematopoiesis that acts upstream of NF-E2 [46]. IRF2 was recently established as a transcriptional regulator of erythropoiesis that controls gene expression through adult erythroid enhancers [20]. Binding sites for NRF2, a transcription factor closely related to NF-E2, are also enriched near hypomethylated CpGs in adult erythroblasts. NRF2 is a transcriptional activator of the antioxidant response and its pharmacological induction in K562 cells results in increased HbF production [47]. The most enriched motif in adult erythroblasts belongs to NF1, a family of transcription factors composed of NFIA, NFIB, NFIC, and NFIX. Several differentially methylated CpGs are located near *NFIA*, *NIFC*, and *NFIX* (Additional file 2: Table S2), suggesting that the expression of these transcription factors, as well as their target genes, may be developmentally regulated by DNA methylation during erythropoiesis. Over-expression of *NFIA* in CD34+ cells leads to increased *HBB* gene expression [48].

### DNA methylation and genetic variation control *HBG2* expression

Treatment with DNA demethylating agents like 5-azacytidine induces HbF production in primates and humans [49,50]. The mechanism implies demethylation of the *HBG2* promoter, directly or indirectly through an effect on cellular stress, that results in an increased synthesis of γ-globin chains [11,12]. However, it is unknown whether changes in DNA methylation at loci unlinked to the *β-globin* cluster on chromosome 11 can also influence the production of HbF in humans. To explore this hypothesis, we tested if common DNA sequence variants associated with HbF production in humans are also associated with changes in DNA methylation levels, that is if they are methylation quantitative trait loci (meQTL). Indeed, recent findings suggest that SNPs associated with complex diseases or traits may exert their phenotypic effect by altering DNA methylation profiles [51,52].

We only analyzed associations between HbF-associated SNPs and DNA methylation levels at the *BCL11A* (chr2:60,678,302-60,781,633), *HBS1L-MYB* (chr6:135,281,517-135,540,311), and *β-globin* (chr11:5,246,696- 5,527,882) loci in adult erythroblasts. We did not include fetal erythroblasts in this analysis because we reasoned that these SNPs affect HbF production in adult erythroid cells. We complemented the 450 k DNA methylation data with measures of DNA methylation at CpGs in the *HBG2* promoter obtained using the targeted assay described above (Additional file 1: Figure S3). We also measured by quantitative PCR the expression of *HBG2* and *HBB* in the same adult cells. In our small sample size (N = 12), we did not identify HbF-associated SNPs that are significantly associated with DNA methylation after accounting for the number of tests performed (Additional file 5: Table S8). Focusing on the *β-globin* locus, we noted that increased DNA methylation in the *HBG2* promoter was associated with a decreased *HBG2*/*HBB* gene expression ratio, as expected (Table 2). When we included in the prediction model both DNA methylation levels at the *HBG2* promoter and genotypes at rs3759074, both terms were nominally associated in the expected direction with the *HBG2/HBB* expression ratio (Table 2). rs3759074 is in linkage disequilibrium with the rs7482144-*Xmn*I variant (*r*^2^ = 0.74) and falls within the *BCL11A* binding site identified in a functional element important for HbF silencing [53]. In a mouse model, inactivation of *BCL11A* and treatment with demethylating 5-aza-2’-deoxycytidine has a synergistic effect on HbF production [21]. Together with these observations, our results suggest that changes in DNA methylation at the *HBG2* promoter act partly independently from genotypes at the *β-globin* locus to control HbF production.

**Table 2:** **Genetic and epigenetic control of** ***HBG2*** **/** ***HBB*** **expression in adult erythroblasts**

**Model**	**rs3759074-A**		**CpG (chr11:5,276,172)**	
	**BETA (SE)**	***P***	**BETA (SE)**	***P***
Model 1: log(*HBG2*/*HBB*) ~ rs3759074	0.98 (0.42)	0.04	-	-
Model 2: log(*HBG2*/*HBB*) ~ CpG	-	-	−6.1 (2.0)	0.01
Model 3: log(*HBG2*/*HBB*) ~ rs3759074 + CpG	5.4 (1.6)	0.008	−0.81 (0.30)	0.02

The A-allele at rs3759074 (chr11:5,257,778) is associated with increased fetal hemoglobin production by genome-wide association studies. In adult erythroblasts, rs3759074-A is associated with increased expression of *HBG2*/*HBB* (Model 1). DNA methylation at a CpG located in the *HBG2* promoter (chr11:5,276,172) is inversely correlated with *HBG2*/*HBB* expression (Model 2). Both genotypes at rs3759074 and DNA methylation at CpG (chr11:5,276,172) are independent predictors of *HBG2*/*HBB* expression levels (Model 3). For this analysis, we used adult erythroblasts from 12 donors. Within this dataset, the rs3759074-A allele frequency is 27% and the mean DNA methylation value at the tested CpG is 61 ± 8%. BETA: arbitrary gene expression units; SE: Standard error.

## Conclusions

We generated comprehensive maps of DNA methylation in human erythroblasts differentiated *ex vivo* from fetal liver or bone marrow CD34+ progenitor cells. At single base pair resolution, we identified 5,937 differentially methylated CpGs that capture many of the transcriptional differences - in terms of transcriptional enhancers and transcription factor binding - between fetal- and adult-stage cells. These analyses also revealed multiple regions that could be of importance for HbF regulation, as indicated by the enrichment of SNPs strongly associated with HbF levels within erythroid enhancers. Most of the differentially methylated CpGs are hypermethylated in fetal erythroblasts. We can explore further these DNA methylation differences to understand what distinguishes fetal from adult erythroblasts during human erythropoiesis.

Our study has some limitations. First, the technology that we used to measure DNA methylation does not distinguish between methylation (5-methylcytosine, 5-mC) and hydroxymethylation (5-hydroxymethylcytosine, 5-hmC). This may be important to functionally explore as 5-mC and 5-hmC have different reported roles in the context of gene regulation [54]. Second, some of the differences observed in DNA methylation levels between fetal and adult erythroblast may be due to slight differences in their growth kinetics. However, we expect this number to be low since our cell morphology and gene expression analyses indicate that these cells are largely undistinguishable. Finally, our analysis of the effect of genetic variation and DNA methylation on globin gene expression is limited by our small sample size. Although our results are consistent with the literature, validation in independent samples is needed to confirm our additive model.

Clinically, one of the most important features of these fetal and adult erythroid cells is their respective production of HbF or HbA. In patients with sickle cell disease or β-thalassemia, increasing HbF production improves disease outcomes. In clinical trials, DNA demethylating agents have shown modest efficacy in increasing HbF production in patients [49,50]. On the other hand, work in a sickle cell mouse model has shown that *BCL11A*-mediated repression and 5-aza-2’-deoxycytidine treatment synergistically control HbF production and improve hematological parameters [21]. This is consistent with our observation that a SNP within a *BCL11A* binding site in a key regulatory element at the *β-globin* locus and DNA methylation are independent predictors of *HBG2* expression in adult erythroblasts. Together, the mouse and human erythroblast results suggest that a combined strategy to inactivate *BCL11A* and promote *HBG2* demethylation may provide the robust induction of HbF production necessary to treat β-hemoglobinopathy patients.

## Additional files

Additional file 1:
**Supplemental data.** Supplementary **Figures S1 to S8**, and supplementary **Tables S1**, **S5**, **S6**, **and S7**. Additional file 2: Table S2. Differentially methylated CpGs between *ex vivo* differentiated fetal and adult erythroblasts. Additional file 3: Table S3. Genes, promoters, and erythroid enhancers that are differentially methylated when comparing fetal and adult erythroblasts. Additional file 4: Table S4. Pathways enriched in differentially methylated genes. Additional file 5: Table S8. Association analysis of HbF-associated SNPs with DNA methylation at the *HBB*, *BCL11A*, and *MYB* loci.

## Abbreviations

BM: Erythroblasts derived from bone marrow
FL: Erythroblasts derived from fetal-liver
GO: Gene ontology
HbA: Adult hemoglobin
HbF: Fetal hemoglobin
MALDI-TOF: Matrix-assisted laser desorption/ionization time-of-flight mass spectrometer
meQTL: Methylation quantitative trait locus
miRNA: micro-RNA
qPCR: Quantitative polymerase chain reaction
SNP: Single nucleotide polyphormism
