# Supplementary material for: Comparison of DNA methylation profiles in human fetal and adult red blood cell progenitors
Source: Genome Med. 2015 Jan 20;7(1):1. doi: 10.1186/s13073-014-0122-2 (PMC4298057; doi:10.1186/s13073-014-0122-2)
Supplement: Additional file 1: — Supplemental data. Supplementary Figures S1 to S8, and supplementary Tables S1, S5, S6, and S7. [file 13073_2014_122_MOESM1_ESM.docx]

**SUPPLEMENTAL DATA**

**Comparison of DNA methylation profiles in human**

**fetal and adult red blood cell progenitors**

Samuel Lessard^1,2^, Mélissa Beaudoin^1^, Karim Benkirane^3^, Guillaume Lettre^1,2^

^1^Montreal Heart Institute, 5000 Bélanger Street, Montréal, Quebec, H1T 1C8, Canada.

^2^Faculté de Médecine, Université de Montréal, 2900 Boul. Édouard-Montpetit, Montréal, Québec, H3T 1J4, Canada.

^3^Hôpital Maisonneuve-Rosemont, 5415 Boul. de l’Assomption, Montréal, Québec, H1T 2M4, Canada.

**Figure S1. *Ex vivo* differentiation of human CD34+ cells**. (**A**) Primary human fetal and adult CD34+ cells are cultured in the expansion medium until day 6 (black arrow). They are then transferred to the differentiation medium until cells are collected at day 18. The medium is changed every 3 days. Consistent with differentiation into erythroblasts, cell size decreases during differentiation. (**B**) Differentiating human fetal and adult CD34+ cells have similar growth curves. (**C**) Wright-Giemsa coloration of CD34+ cells during differentiation shows the expected change in morphology (60X): at day 6, cells have myeloid stem cell morphology and at day 18, cells of both types display either orthochromatic or polychromatic erythroblast morphology. FL: CD34+ cells from fetal liver; BM: CD34+ cells from bone marrow (adult).

**Figure S2. *Ex vivo* differentiated CD34+ cells exhibit gene expression and hemoglobin production that are characteristic of fetal or adult erythroblasts.** (**A**) Bone marrow-derived erythroblasts express significantly more of the stage-specific γ-globin transcriptomic repressor *BCL11A* as assessed by qPCR. (**B**) Fetal liver-derived erythroblasts cells express significantly less of the *KLF1* gene, which has been show to preferentially activate the *HBB* gene as well as promoting γ-globin repression. For A and B, we calculated *P*-values with Student’s *t*-test. (**C**) Erythroblasts from bone marrow produce more adult hemoglobin (HbA), whereas fetal liver-derived erythroblasts produce exclusively fetal hemoglobin (HbF). Error bars are ± SEM. (**D-E**) Representative examples of capillary electrophoresis chromatograms used to quantify hemoglobin in erythroblasts differentiated from bone marrow (BM) or fetal liver (FL) CD34+ cells.

**Figure S3. DNA methylation in the *HBG2* (*γ-globin*) promoter.** CpGs in the *HBG2* promoter are hypomethylated and hypermethylated in fetal and adult erythroblasts, respectively. Blue: erythroblasts from bone marrow. Red: erythroblasts from fetal liver. Error bars are ± SEM.

**
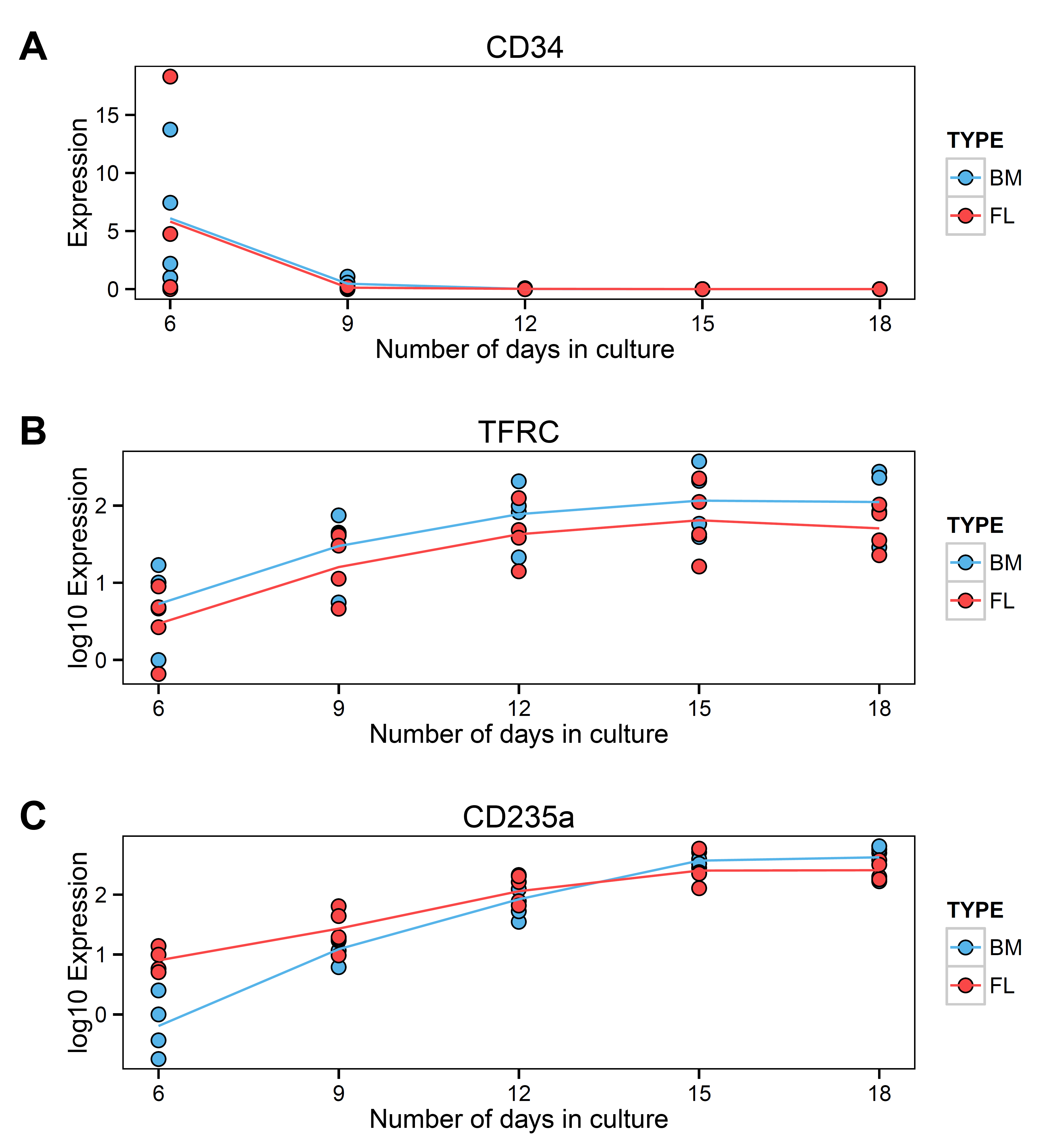
**

**Figure S4. RNA expression of cell surface markers during *ex vivo* differentiation.** Relative expression values were obtained by qPCR. (**A**) *CD34* expression decreases during differentiation. (**B**) Expression of *TFRC* (transferrin receptor) increases during differentiation. (**C**) *CD235a* (glycophorin A) expression increases during differentiation. No significant differences were observed between fetal and adult-stage cells for all markers (*t*-test, all *P* > 0.15), expect for *CD235a* at day 6 (*t*-test, *P*=0.03).

**Figure S5. DNA methylation data normalization.** DNA methylation β-value distributions across all samples before (**A**) and after (**B**) normalization with ARRm and the removal of probes that contain DNA polymorphisms. Red: fetal liver (FL)-derived erythroblasts; Blue: bone marrow (BM)-derived erythroblasts.

**Figure S6. Correlations of DNA methylation between samples of our study and samples from the Roadmap Epigenomics Project. (A)** Correlation between DNA methylation values from our study (adult erythroblasts from 12 donors) and mobilized adult CD34+ cells from the Roadmap Epigenomics Project. For this analysis, we analyzed 80,136 CpGs with DNA methylation values present in both datasets. The correlation is strong (Pearson’s *r*^2^=0.83, *P*<2.2x10^-16^), thus validating DNA methylation values generated with the Illumina HumanMethylation 450k BeadChip. **(B)** Correlation coefficients (Pearson’s *r*^2^) for DNA methylation levels in fetal- (FL) and adult-stage (BM) erythroblasts when compared to reduced representation bisulfite sequencing data from different cell types and tissues from the Roadmap Epigenomics project. We restricted this analysis to CpGs common across all cell types (*i.e.* a subset of the CpGs included in (A)), which explains the difference in *r*^2^ between (A) and (B).

**Figure S7. Relationship between DNA methylation and the expression of *GCNT2* isoforms. (A)** DNA methylation levels at cg22187251 (isoform A exon), cg12695465 (isoform B exon), and cg14322298 (isoform C exon). **(B)** Expression of the different isoforms reported by Cufflinks. **(C)** and **(D)** Relative expression of the *GCNT2* isoforms (same color codes as in (B)) in adult (C) and fetal (D) erythroblasts.

**Figure S8**. **Enrichment of positive and negative correlations of DNA methylation and expression in genes, promoters, and erythroid enhancers.** We calculated the correlations between DNA methylation at CpGs in genes, promoters and enhancers with the expression of the corresponding genes. For enhancers, the closest gene was used (maximum distance of 1Mb). We report the ratio of positive and negative correlations (Pearson’s coefficient) with *P*<1x10^-4^.

**Table S1. List of primers used for quantitative PCR analyses.** All primer sequences are 5'-3'.

| **Target** | **Sense primer** | **Antisense primer** |
| --- | --- | --- |
| *BCL11A-XL* | TCAAATAGCACTTGACTCTGCCTG | CAGCATTCTTGCAACTTTTCCC |
| *KLF1* | GGCAAGAGCTACACCAAGAG | TGTGTTTCCGGTAGTGGC |
| *HBG2* | TGGATGATCTCAAGGGCAC | TCAGTGGTATCTGGAGGACA |
| *HBB* | CTGAGGAGAAGTCTGCCGTTA | AGCATCAGGAGTGGACAGAT |
| *CD235a* | GGCTAAGGTCAGACACTGAC | TGTGCATTGCCACCTCAGTG |
| *CD34* | AATGAGGCCACAACAAACATCACA | CTGTCCTTCTTAACCTCCGCACAGC |
| *TFRC* | AAAATCCGGTGTAGGCACAG | CCTTTAAATGCAGGGACG |
| *GADPH* | GACAGTCAGCCGCATCTTC | GCAACAATATCCACTTTACCAGAG |

**Table S2.** (Excel file .xls). **Annotated list of differentially methylated CpGs.**

This table is available in a separate Excel file.

**Table S3.** (Excel file .xls). **Annotated list of differentially methylated genes, promoters and enhancers.**

This table is available in a separate Excel file.

**Table S4.** (Excel file .xls). This enrichment analysis was carried out with DAVID [1, 2] using default parameters and pathways. We searched for enriched pathways in genes that display a mean β-value difference of at least 0.1 in their body or promoter, and a significant combined *P*<9x10^-7^. We used all genes captured by the 450K Illumina array as background. We report pathways with an enrichment *P*<0.1 in a separate Excel file (**Additional File 4**).

**Table S5.** **Pathway clusters enriched in differentially methylated genes.** Using the DAVID clustering tool [1, 2], we grouped similar pathways with an enrichment *P*<0.05. We report clusters in which at least one pathway was significant (Benjamini-corrected *P*<0.05). The “Best pathway” column represents the most significant pathway in each of the highlighted clusters.

| **Cluster** | **Number of pathways** | **Score** | **Best pathway** | **Best P-value** | **Corrected P-value (Benjamini)** |
| --- | --- | --- | --- | --- | --- |
| Regulation | 31 | 4.38 | Signal peptide | 2.7x10^-9^ | 7.7x10^-6^ |
| Healing | 12 | 2.68 | Response to wounding | 6.6x10^-8^ | 9.1x10^-5^ |
| Sodium transport | 6 | 2.46 | Sodium | 1.0x10^-3^ | 0.046 |
| Oxygen | 8 | 2.46 | Dioxygenase | 7.34x10^-4^ | 0.044 |
| Immunity and cytokine production | 221 | 2.38 | Immune response | 3.4x10^-12^ | 9.31x10^-9^ |
| Body fluid/ circulation | 12 | 2.00 | Regulation of body fluid levels | 1.56x10^-4^ | 0.038 |

**Table S6. Transcription factor binding sites that are enriched near CpGs that are hypomethylated in fetal erythroblasts.** The enrichment analysis was carried out with the HOMER software [3].

| **Motif Name** | **Consensus** | **P-value** | **q-value (Benjamini)** | **# of target sequences with motif (out of 5000)(%)** | **# of background sequences with motif (out of 4773)(%)** |
| --- | --- | --- | --- | --- | --- |
| Sox2(HMG)/mES-Sox2-ChIP-Seq/Homer | BCCATTGTTC | 1.00E-13 | 0 | 517 (10.3) | 354.6 (7.4) |
| Tcf3(HMG)/mES-Tcf3-ChIP-Seq/Homer | ASWTCAAAGG | 1.00E-08 | 0 | 148 (3) | 83.9 (1.8) |
| REST-NRSF(Zf)/Jurkat-NRSF-ChIP-Seq/Homer | GGMGCTGTCCATGGTGCTGA | 1.00E-08 | 0 | 18 (0.4) | 3.3 (0.1) |
| Sox6(HMG)/Myotubes-Sox6-ChIP-Seq(GSE32627)/Homer | CCATTGTTNY | 1.00E-08 | 0 | 833 (16.7) | 659.8 (13.8) |
| GATA-DR4(Zf)/iTreg-Gata3-ChIP-Seq(GSE20898)/Homer | AGATGKDGAGATAAG | 1.00E-05 | 0.0001 | 39 (0.8) | 16.4 (0.3) |
| Maz(Zf)/HepG2-Maz-ChIP-Seq(GSE31477)/Homer | GGGGGGGG | 1.00E-05 | 0.0002 | 1684 (33.7) | 1468.6 (30.8) |
| Tcf4(HMG)/Hct116-Tcf4-ChIP-Seq/Homer | ASATCAAAGGVA | 1.00E-05 | 0.0002 | 244 (4.9) | 175.7 (3.7) |
| FOXA1(Forkhead)/LNCAP-FOXA1-ChIP-Seq/Homer | WAAGTAAACA | 1.00E-05 | 0.0002 | 527 (10.5) | 419 (8.8) |
| HNF6(Homeobox)/Liver-Hnf6-ChIP-Seq(ERP000394) | NTATYGATCH | 1.00E-04 | 0.0004 | 228 (4.6) | 164.3 (3.4) |
| TCFL2(HMG)/K562-TCF7L2-ChIP-Seq(GSE29196)/Homer | ACWTCAAAGG | 1.00E-04 | 0.0005 | 50 (1) | 25.9 (0.5) |
| Sox3(HMG)/NPC-Sox3-ChIP-Seq(GSE33059)/Homer | CCWTTGTY | 1.00E-04 | 0.0006 | 866 (17.3) | 727.7 (15.3) |
| Tcfcp2l1(CP2)/mES-Tcfcp2l1-ChIP-Seq/Homer | NRAACCRGTTYRAACCRGYT | 1.00E-04 | 0.0011 | 96 (1.9) | 60.3 (1.3) |
| GAGA-repeat/Arabidopsis-Promoters/Homer | CTCTCTCTCY | 1.00E-04 | 0.0012 | 879 (17.6) | 743.5 (15.6) |
| Unknown2/Drosophila-Promoters/Homer | CATCMCTA | 1.00E-04 | 0.0013 | 825 (16.5) | 695.2 (14.6) |
| Foxa2(Forkhead)/Liver-Foxa2-ChIP-Seq/Homer | CYTGTTTACWYW | 1.00E-03 | 0.0023 | 356 (7.1) | 280.5 (5.9) |
| PPARE(NR/DR1)/3T3L1-Pparg-ChIP-Seq/Homer | TGACCTTTGCCCCA | 1.00E-03 | 0.0032 | 637 (12.7) | 531.7 (11.2) |
| JunD(bZIP)/K562-JunD-ChIP-Seq/Homer | ATGACGTCATCN | 1.00E-03 | 0.0035 | 42 (0.8) | 22.9 (0.5) |
| Srebp2(HLH)/HepG2-Srebp2-ChIP-Seq/Homer | CGGTCACSCCAC | 1.00E-03 | 0.0044 | 97 (1.9) | 64.8 (1.4) |
| CRX(Homeobox)/Retina-Crx-ChIP-Seq/Homer | GCTAATCC | 1.00E-03 | 0.0045 | 1197 (23.9) | 1046.5 (21.9) |
| Oct4(POU/Homeobox)/mES-Oct4-ChIP-Seq/Homer | ATTTGCATAW | 1.00E-03 | 0.0046 | 208 (4.2) | 156.4 (3.3) |
| Lhx3-like?(Homeobox)/Forebrain-p300-ChIP-Seq/Homer | CTAATTAGCH | 1.00E-03 | 0.0046 | 486 (9.7) | 399.7 (8.4) |
| GATA-IR4(Zf)/iTreg-Gata3-ChIP-Seq(GSE20898)/Homer | NAGATWNBNATCTNN | 1.00E-03 | 0.0046 | 34 (0.7) | 17.5 (0.4) |
| Nr5a2(NR)/Pancreas-LRH1-ChIP-Seq(GSE34295)/Homer | BTCAAGGTCA | 1.00E-03 | 0.0048 | 422 (8.4) | 343.8 (7.2) |
| NFAT(RHD)/Jurkat-NFATC1-ChIP-Seq/Homer | ATTTTCCATT | 1.00E-03 | 0.0048 | 449 (9) | 367.5 (7.7) |
| Oct2(POU/Homeobox)/Bcell-Oct2-ChIP-Seq/Homer | ATATGCAAAT | 1.00E-03 | 0.0057 | 130 (2.6) | 92.2 (1.9) |
| Nr5a2(NR)/mES-Nr5a2-ChIP-Seq/Homer | BTCAAGGTCA | 1.00E-03 | 0.0061 | 342 (6.8) | 274.5 (5.8) |
| Esrrb(NR)/mES-Esrrb-ChIP-Seq/Homer | KTGACCTTGA | 1.00E-03 | 0.0088 | 366 (7.3) | 297.8 (6.2) |
| Nanog(Homeobox)/mES-Nanog-ChIP-Seq/Homer | RGCCATTAAC | 1.00E-02 | 0.0116 | 2132 (42.7) | 1934.7 (40.6) |
| RXR(NR/DR1)/3T3L1-RXR-ChIP-Seq/Homer | TAGGGCAAAGGTCA | 1.00E-02 | 0.0124 | 780 (15.6) | 673.3 (14.1) |
| BORIS(Zf)/K562-CTCFL-ChIP-Seq/Homer | CNNBRGCGCCCCCTGSTGGC | 1.00E-02 | 0.014 | 247 (4.9) | 195.6 (4.1) |
| Eomes(T-box)/H9-Eomes-ChIP-Seq/Homer | AGGTGTTAAN | 1.00E-02 | 0.014 | 1705 (34.1) | 1534.6 (32.2) |
| FOXA1(Forkhead)/MCF7-FOXA1-ChIP-Seq/Homer | WAAGTAAACA | 1.00E-02 | 0.0149 | 416 (8.3) | 345.7 (7.3) |
| CHR/Cell-Cycle-Exp/Homer | SRGTTTCAAA | 1.00E-02 | 0.0188 | 268 (5.4) | 215.5 (4.5) |
| PCF/Arabidopsis-Promoters/Homer | NNWWWTGGGCYTDDN | 1.00E-02 | 0.0215 | 108 (2.2) | 78.4 (1.6) |
| Gata1(Zf)/K562-GATA1-ChIP-Seq/Homer | SAGATAAGRV | 1.00E-02 | 0.0216 | 354 (7.1) | 292.5 (6.1) |
| Gata4(Zf)/Heart-Gata4-ChIP-Seq(GSE35151)/Homer | NBWGATAAGR | 1.00E-02 | 0.0216 | 545 (10.9) | 464.6 (9.7) |
| NFY(CCAAT)/Promoter/Homer | RGCCAATSRG | 1.00E-02 | 0.0217 | 414 (8.3) | 346.8 (7.3) |
| PAX3:FKHR-fusion(Paired/Homeobox)/Rh4-PAX3:FKHR-ChIP-Seq/Homer | ACCRTGACTAATTNN | 1.00E-02 | 0.0217 | 76 (1.5) | 52.7 (1.1) |
| GFY-Staf/Promoters/Homer | RACTACAATTCCCAGAAKGC | 1.00E-02 | 0.0247 | 22 (0.4) | 12 (0.3) |
| p53(p53)/p53-ChIP-Chip/Homer | NRGACATGTCYRGACATGTC | 1.00E-02 | 0.0278 | 5 (0.1) | 1.7 (0) |
| AP-2alpha(AP2)/Hela-AP2alpha-ChIP-Seq/Homer | ATGCCCTGAGGC | 1.00E-02 | 0.0418 | 910 (18.2) | 805.3 (16.9) |
| PRDM14(Zf)/H1-PRDM14-ChIP-Seq/Homer | RGGTCTCTAACY | 1.00E-02 | 0.0478 | 187 (3.7) | 149.8 (3.1) |

**Table S7. Transcription factor binding sites that are enriched near CpGs that are hypomethylated in adult erythroblasts.** The enrichment analysis was carried out with the HOMER software [3].

| **Motif Name** | **Consensus** | **P-value** | **q-value (Benjamini)** | **# of target sequences with motif (out of 5000)(%)** | **# of background sequences with motif (out of 4986)(%)** |
| --- | --- | --- | --- | --- | --- |
| NF1(CTF)/LNCAP-NF1-ChIP-Seq/Homer | CYTGGCABNSTGCCAR | 1.00E-211 | 0 | 722 (14.4) | 184 (3.7) |
| IRF2(IRF)/Erythroblas-IRF2-ChIP-Seq(GSE36985)/Homer | GAAASYGAAASY | 1.00E-78 | 0 | 155 (3.1) | 21.3 (0.4) |
| Tlx?/NPC-H3K4me1-ChIP-Seq/Homer | CTGGCAGSCTGCCA | 1.00E-76 | 0 | 563 (11.3) | 237.3 (4.8) |
| NF1-halfsite(CTF)/LNCaP-NF1-ChIP-Seq/Homer | YTGCCAAG | 1.00E-56 | 0 | 1627 (32.5) | 1132.1 (22.7) |
| ISRE(IRF)/ThioMac-LPS-exp/HOMER | AGTTTCASTTTC | 1.00E-54 | 0 | 106 (2.1) | 14.2 (0.3) |
| Bach1(bZIP)/K562-Bach1-ChIP-Seq(GSE31477)/Homer | AWWNTGCTGAGTCAT | 1.00E-42 | 0 | 91 (1.8) | 14.3 (0.3) |
| RUNX1(Runt)/Jurkat-RUNX1-ChIP-Seq/Homer | AAACCACARM | 1.00E-37 | 0 | 832 (16.6) | 530.7 (10.6) |
| RUNX2(Runt)/PCa-RUNX2-ChIP-Seq(GSE33889)/Homer | NWAACCACADNN | 1.00E-36 | 0 | 665 (13.3) | 398.4 (8) |
| Nrf2(bZIP)/Lymphoblast-Nrf2-ChIP-Seq(GSE37589)/Homer | HTGCTGAGTCAT | 1.00E-36 | 0 | 81 (1.6) | 13.9 (0.3) |
| Jun-AP1(bZIP)/K562-cJun-ChIP-Seq/Homer | GATGASTCATCN | 1.00E-33 | 0 | 273 (5.5) | 119.4 (2.4) |
| RUNX-AML(Runt)/CD4+-PolII-ChIP-Seq/Homer | GCTGTGGTTW | 1.00E-30 | 0 | 617 (12.3) | 380.3 (7.6) |
| NF-E2(bZIP)/K562-NFE2-ChIP-Seq/Homer | GATGACTCAGCA | 1.00E-29 | 0 | 94 (1.9) | 22.4 (0.5) |
| AP-1(bZIP)/ThioMac-PU.1-ChIP-Seq/Homer | VTGACTCATC | 1.00E-29 | 0 | 588 (11.8) | 362.7 (7.3) |
| RUNX(Runt)/HPC7-Runx1-ChIP-Seq/Homer | SAAACCACAG | 1.00E-27 | 0 | 616 (12.3) | 391.2 (7.8) |
| EWS:FLI1-fusion(ETS)/SK_N_MC-EWS:FLI1-ChIP-Seq/Homer | VACAGGAAAT | 1.00E-26 | 0 | 585 (11.7) | 368.8 (7.4) |
| ERG(ETS)/VCaP-ERG-ChIP-Seq/Homer | ACAGGAAGTG | 1.00E-25 | 0 | 1485 (29.7) | 1156.6 (23.2) |
| HIF1b(HLH)/O785-HIF1b-ChIP-Seq(GSE34871)/Homer | VTGASTCABH | 1.00E-25 | 0 | 498 (10) | 303.8 (6.1) |
| Fli1(ETS)/CD8-FLI-ChIP-Seq(GSE20898)/Homer | NRYTTCCGGH | 1.00E-17 | 0 | 1102 (22) | 859.7 (17.2) |
| ETV1(ETS)/GIST48-ETV1-ChIP-Seq/Homer | AACCGGAAGT | 1.00E-16 | 0 | 1253 (25.1) | 1009.4 (20.2) |
| EWS:ERG-fusion(ETS)/CADO_ES1-EWS:ERG-ChIP-Seq/Homer | ATTTCCTGTN | 1.00E-15 | 0 | 669 (13.4) | 489.5 (9.8) |
| Stat3+il23(Stat)/CD4-Stat3-ChIP-Seq/Homer | SVYTTCCNGGAARB | 1.00E-14 | 0 | 527 (10.5) | 373.5 (7.5) |
| SPDEF(ETS)/VCaP-SPDEF-ChIP-Seq/Homer | ASWTCCTGBT | 1.00E-09 | 0 | 813 (16.3) | 655.6 (13.1) |
| STAT4(Stat)/CD4-Stat4-ChIP-Seq/Homer | NYTTCCWGGAAR | 1.00E-09 | 0 | 641 (12.8) | 502.5 (10.1) |
| ARE(NR)/LNCAP-AR-ChIP-Seq/Homer | RGRACASNSTGTYCYB | 1.00E-09 | 0 | 179 (3.6) | 109.9 (2.2) |
| IRF4(IRF)/GM12878-IRF4-ChIP-Seq/Homer | ACTGAAACCA | 1.00E-09 | 0 | 294 (5.9) | 203.6 (4.1) |
| GFX(?)/Promoter/Homer | ATTCTCGCGAGA | 1.00E-09 | 0 | 12 (0.2) | 0.5 (0) |
| Elk1(ETS)/Hela-Elk1-ChIP-Seq(GSE31477)/Homer | HACTTCCGGY | 1.00E-08 | 0 | 485 (9.7) | 370.4 (7.4) |
| PRDM1/BMI1(Zf)/Hela-PRDM1-ChIP-Seq(GSE31477)/Homer | ACTTTCACTTTC | 1.00E-08 | 0 | 380 (7.6) | 279.6 (5.6) |
| T1ISRE(IRF)/Ifnb-Exp/Homer | ACTTTCGTTTCT | 1.00E-08 | 0 | 18 (0.4) | 3.2 (0.1) |
| Usf2(HLH)/C2C12-Usf2-ChIP-Seq(GSE36030)/Homer | GTCACGTGGT | 1.00E-07 | 0 | 247 (4.9) | 170.7 (3.4) |
| Elk4(ETS)/Hela-Elk4-ChIP-Seq(GSE31477)/Homer | NRYTTCCGGY | 1.00E-07 | 0 | 508 (10.2) | 396.1 (7.9) |
| ETS1(ETS)/Jurkat-ETS1-ChIP-Seq/Homer | ACAGGAAGTG | 1.00E-07 | 0 | 933 (18.7) | 786.1 (15.8) |
| GABPA(ETS)/Jurkat-GABPa-ChIP-Seq/Homer | RACCGGAAGT | 1.00E-07 | 0 | 793 (15.9) | 658.8 (13.2) |
| CEBP(bZIP)/CEBPb-ChIP-Seq/Homer | ATTGCGCAAC | 1.00E-06 | 0 | 384 (7.7) | 293.4 (5.9) |
| HLH-1(bHLH)/cElegans-Embryo-HLH1-ChIP-Seq(modEncode)/Homer | RACAGCTGTTBH | 1.00E-06 | 0 | 543 (10.9) | 440.4 (8.8) |
| GRE(NR/IR3)/A549-GR-ChIP-Seq/Homer | NRGVACABNVTGTYCY | 1.00E-06 | 0 | 97 (1.9) | 57.6 (1.2) |
| MafK(bZIP)/C2C12-MafK-ChIP-Seq(GSE36030)/Homer | GCTGASTCAGCA | 1.00E-05 | 0 | 168 (3.4) | 114.7 (2.3) |
| n-Myc(HLH)/mES-nMyc-ChIP-Seq/Homer | VRCCACGTGG | 1.00E-05 | 0 | 508 (10.2) | 414.3 (8.3) |
| Nkx2.5(Homeobox)/HL1-Nkx2.5.biotin-ChIP-Seq/Homer | RRSCACTYAA | 1.00E-05 | 0 | 1403 (28.1) | 1257.5 (25.2) |
| GRE/RAW264.7-GRE-ChIP-Seq/Homer | VAGRACAKWCTGTYC | 1.00E-05 | 0 | 152 (3) | 104.8 (2.1) |
| Bcl6(Zf)/Liver-Bcl6-ChIP-Seq(GSE31578)/Homer | NNNCTTTCCAGGAAA | 1.00E-05 | 0 | 861 (17.2) | 747.8 (15) |
| ETS:E-box/HPC7-Scl-ChIP-Seq/Homer | AGGAARCAGCTG | 1.00E-05 | 0 | 85 (1.7) | 51.9 (1) |
| SFP1/SacCer-Promoters/Homer | DDAAAAATTTTY | 1.00E-05 | 0.0001 | 51 (1) | 26.3 (0.5) |
| c-Myc(HLH)/LNCAP-cMyc-ChIP-Seq/Homer | VCCACGTG | 1.00E-05 | 0.0001 | 402 (8) | 324.5 (6.5) |
| CTCF-SatelliteElement/CD4+-CTCF-ChIP-Seq/Homer | TGCAGTTCCMVNWRTGGCCA | 1.00E-04 | 0.0001 | 8 (0.2) | 0.7 (0) |
| Max(HLH)/K562-Max-ChIP-Seq/Homer | RCCACGTGGYYN | 1.00E-04 | 0.0002 | 434 (8.7) | 357.9 (7.2) |
| Max(HLH)/K562-Max-ChIP-Seq/Homer | RCCACGTGGYYN | 1.00E-04 | 0.0002 | 434 (8.7) | 357.9 (7.2) |
| bHLHE40(HLH)/HepG2-BHLHE40-ChIP-Seq/Homer | SGKCACGTGM | 1.00E-04 | 0.0002 | 132 (2.6) | 92 (1.8) |
| Ets1-distal(ETS)/CD4+-PolII-ChIP-Seq/Homer | MACAGGAAGT | 1.00E-04 | 0.0003 | 284 (5.7) | 224.8 (4.5) |
| STAT1(Stat)/HelaS3-STAT1-ChIP-Seq/Homer | NATTTCCNGGAAAT | 1.00E-04 | 0.0003 | 211 (4.2) | 160.5 (3.2) |
| PU.1-IRF/Bcell-PU.1-ChIP-Seq/Homer | MGGAAGTGAAAC | 1.00E-04 | 0.0004 | 905 (18.1) | 803.4 (16.1) |
| Atf1(bZIP)/K562-ATF1-ChIP-Seq(GSE31477)/Homer | GATGACGTCA | 1.00E-03 | 0.0006 | 404 (8.1) | 336.9 (6.8) |
| PIF5ox(bHLH)/Arabidopsis-PIF5ox-ChIP-Seq(GSE35062)/Homer | BCACGTGVDN | 1.00E-03 | 0.0007 | 666 (13.3) | 580.2 (11.6) |
| Tbx5(T-box)/HL1-Tbx5.biotin-ChIP-Seq/Homer | AGGTGTCA | 1.00E-03 | 0.001 | 2023 (40.5) | 1897.7 (38) |
| Mef2a(MADS)/HL1-Mef2a.biotin-ChIP-Seq/Homer/ | CYAAAAATAG | 1.00E-03 | 0.0011 | 239 (4.8) | 189.4 (3.8) |
| E-box/Arabidopsis-Promoters/Homer | GCCACGTG | 1.00E-03 | 0.0011 | 355 (7.1) | 294.1 (5.9) |
| EBNA1(EBV virus)/Raji-EBNA1-ChIP-Seq(GSE30709)/Homer | GGYAGCAYDTGCTDCCCNNN | 1.00E-03 | 0.0014 | 11 (0.2) | 3.4 (0.1) |
| Cbf1(bHLH)/Yeast-Cbf1-ChIP-Seq(GSE29506)/Homer | TCACGTGAYH | 1.00E-03 | 0.0017 | 210 (4.2) | 165.4 (3.3) |
| PR(NR)/T47D-PR-ChIP-Seq(GSE31130)/Homer | VAGRACAKNCTGTBC | 1.00E-03 | 0.0017 | 1282 (25.6) | 1177.1 (23.6) |
| AR-halfsite(NR)/LNCaP-AR-ChIP-Seq/Homer | CCAGGAACAG | 1.00E-03 | 0.0021 | 2370 (47.4) | 2248.9 (45.1) |
| ZFX(Zf)/mES-Zfx-ChIP-Seq/Homer | AGGCCTRG | 1.00E-03 | 0.0027 | 1123 (22.5) | 1027.3 (20.6) |
| Atoh1(bHLH)/Cerebellum-Atoh1-ChIP-Seq/Homer | VNRVCAGCTGGY | 1.00E-03 | 0.003 | 834 (16.7) | 750.9 (15.1) |
| CRE(bZIP)/Promoter/Homer | CSGTGACGTCAC | 1.00E-02 | 0.0059 | 143 (2.9) | 110.1 (2.2) |
| NeuroD1(bHLH)/Islet-NeuroD1-ChIP-Seq(GSE30298)/Homer | GCCATCTGTT | 1.00E-02 | 0.0059 | 623 (12.5) | 554.4 (11.1) |
| Klf4(Zf)/mES-Klf4-ChIP-Seq/Homer | GCCACACCCA | 1.00E-02 | 0.0064 | 367 (7.3) | 314.4 (6.3) |
| Hoxc9/Ainv15-Hoxc9-ChIP-Seq/Homer | GGCCATAAATCA | 1.00E-02 | 0.0071 | 308 (6.2) | 260 (5.2) |
| ATF3(bZIP)/K562-ATF3-ChIP-Seq/Homer | SGGTCACGTGAC | 1.00E-02 | 0.0083 | 163 (3.3) | 129.7 (2.6) |
| Pho2(bHLH)/Yeast-Pho2-ChIP-Seq(GSE29506)/Homer | CCCACGTGCT | 1.00E-02 | 0.0112 | 355 (7.1) | 306.3 (6.1) |
| HIF2a(HLH)/O785-HIF2a-ChIP-Seq(GSE34871)/Homer | GCACGTACCC | 1.00E-02 | 0.0117 | 331 (6.6) | 284.5 (5.7) |
| HIF-1a(HLH)/MCF7-HIF1a-ChIP-Seq/Homer | TACGTGCV | 1.00E-02 | 0.0143 | 228 (4.6) | 190.1 (3.8) |
| Tal1 | CATCTG | 1.00E-02 | 0.0179 | 925 (18.5) | 853.8 (17.1) |
| E2F1(E2F)/Hela-E2F1-ChIP-Seq/Hoemr | CWGGCGGGAA | 1.00E-02 | 0.0197 | 199 (4) | 165.6 (3.3) |
| LIN-39(Homeobox)/cElegans.L3-LIN39-ChIP-Seq(modEncode)/Homer | ATGATTRATG | 1.00E-02 | 0.0202 | 638 (12.8) | 578.2 (11.6) |
| c-Myc/mES-cMyc-ChIP-Seq/Homer | VVCCACGTGG | 1.00E-02 | 0.0216 | 326 (6.5) | 283.2 (5.7) |
| HOXA9/HSC-Hoxa9-ChIP-Seq(GSE33509)/Homer | GGCCATAAATCA | 1.00E-02 | 0.0222 | 395 (7.9) | 348.7 (7) |
| Pdx1(Homeobox)/Islet-Pdx1-ChIP-Seq/Homer | YCATYAATCA | 1.00E-02 | 0.0222 | 508 (10.2) | 456 (9.1) |
| STAT5(Stat)/mCD4+-Stat5a\|b-ChIP-Seq/Homer | RTTTCTNAGAAA | 1.00E-02 | 0.0239 | 225 (4.5) | 190.6 (3.8) |
| Tbox:Smad/ESCd5-Smad2_3-ChIP-Seq(GSE29422)/Homer | AGGTGHCAGACA | 1.00E-02 | 0.0271 | 122 (2.4) | 97.7 (2) |
| Stat3(Stat)/mES-Stat3-ChIP-Seq/Homer | CTTCCGGGAA | 1.00E-02 | 0.0299 | 347 (6.9) | 305.2 (6.1) |
| p63(p53)/Keratinocyte-p63-ChIP-Seq/Homer | NNDRCATGYCYNRRCATGYH | 1.00E-02 | 0.0315 | 206 (4.1) | 174.4 (3.5) |
| MafF(bZIP)/HepG2-MafF-ChIP-Seq(GSE31477)/Homer | HWWGTCAGCAWWTTT | 1.00E-01 | 0.034 | 152 (3) | 125.3 (2.5) |
| Nkx3.1(Homeobox)/LNCaP-Nkx3.1-ChIP-Seq(GSE28264)/Homer | AAGCACTTAA | 1.00E-01 | 0.035 | 1428 (28.6) | 1352.4 (27.1) |
| CES-1(Homeobox)/cElegans-L1-CES1-ChIP-Seq(modEncode)/Homer | AAATTSAATTTN | 1.00E-01 | 0.0368 | 260 (5.2) | 225.7 (4.5) |
| PBX1(Homeobox)/MCF7-PBX1-ChIP-Seq(GSE28007)/Homer | GSCTGTCACTCA | 1.00E-01 | 0.0368 | 52 (1) | 37.6 (0.8) |
| p53(p53)/mES-cMyc-ChIP-Seq/Homer | ACATGCCCGGGCAT | 1.00E-01 | 0.0368 | 21 (0.4) | 12.2 (0.2) |
| MYB(HTH)/ERMYB-Myb-ChIPSeq(GSE22095)/Homer | GGCVGTTR | 1.00E-01 | 0.0388 | 1171 (23.4) | 1102.3 (22.1) |
| GATA:SCL/Ter119-SCL-ChIP-Seq/Homer | CRGCTGBNGNSNNSAGATAA | 1.00E-01 | 0.0391 | 100 (2) | 79.2 (1.6) |
| PAX5-shortForm(Paired/Homeobox)/GM12878-PAX5-ChIP-Seq/Homer | GTCACGCTCSCTGM | 1.00E-01 | 0.0444 | 78 (1.6) | 60.5 (1.2) |
| USF1(HLH)/GM12878-Usf1-ChIP-Seq/Homer | SGTCACGTGR | 1.00E-01 | 0.0454 | 355 (7.1) | 316.5 (6.3) |

**Table S8.** (Excel file .xls). **Association results between HbF-associated SNPs and DNA methylation at CpGs in the *BCL11A*, *MYB* and *β-globin* loci.**

This table is available in a separate Excel file.**Supplementary** **References**

1. Huang da W, Sherman BT, Lempicki RA: **Bioinformatics enrichment tools: paths toward the comprehensive functional analysis of large gene lists.** *Nucleic acids research* 2009, **37:**1-13.

2. Huang da W, Sherman BT, Lempicki RA: **Systematic and integrative analysis of large gene lists using DAVID bioinformatics resources.** *Nature protocols* 2009, **4:**44-57.

3. Heinz S, Benner C, Spann N, Bertolino E, Lin YC, Laslo P, Cheng JX, Murre C, Singh H, Glass CK: **Simple combinations of lineage-determining transcription factors prime cis-regulatory elements required for macrophage and B cell identities.** *Molecular cell* 2010, **38:**576-589.
